# Glycaemic control targets after traumatic brain injury: a systematic review and meta-analysis

**DOI:** 10.1186/s13054-017-1883-y

**Published:** 2018-01-19

**Authors:** Jeroen Hermanides, Mark P. Plummer, Mark Finnis, Adam M. Deane, Jonathan P. Coles, David K. Menon

**Affiliations:** 1 Division of Anaesthesia, Department of Medicine, University of Cambridge, Addenbrooke’s Hospital, Cambridge, CB2 0QQ UK; 2 Neurosciences Critical Care Unit, Addenbrooke’s Hospital, Cambridge, CB2 0QQ UK; 3 Intensive Care Unit, Royal Adelaide Hospital, Adelaide, 5000 Australia; 4 Intensive Care Unit, Royal Melbourne Hospital, Melbourne, 3050 Australia; 5 Department of Anesthesiology, Academic Medical Centre, Meibergdreef 9, 1105 AZ Amsterdam, The Netherlands

**Keywords:** Traumatic brain injury, Glycaemia, Intensive insulin therapy, Glucose control, Systematic review

## Abstract

**Background:**

Optimal glycaemic targets in traumatic brain injury (TBI) remain unclear. We performed a systematic review and meta-analysis of randomised controlled trials (RCTs) comparing intensive with conventional glycaemic control in TBI requiring admission to an intensive care unit (ICU).

**Methods:**

We systematically searched MEDLINE, EMBASE and the Cochrane Central Register of Controlled Trials to November 2016. Outcomes of interest included ICU and in-hospital mortality, poor neurological outcome, the incidence of hypoglycaemia and infective complications. Data were analysed by pairwise random effects models with secondary analysis of differing levels of conventional glycaemic control.

**Results:**

Ten RCTs, involving 1066 TBI patients were included. Three studies were conducted exclusively in a TBI population, whereas in seven trials, the TBI population was a sub-cohort of a mixed neurocritical or general ICU population. Glycaemic targets with intensive control ranged from 4.4 to 6.7 mmol/L, while conventional targets aimed to keep glucose levels below thresholds of 8.4–12 mmol/L. Conventional versus intensive control showed no association with ICU or hospital mortality (relative risk (RR) (95% CI) 0.93 (0.68–1.27), *P* = 0.64 and 1.07 (0.84–1.36), *P* = 0.62, respectively). The risk of a poor neurological outcome was higher with conventional control (RR (95% CI) = 1.10 (1.001–1.24), *P* = 0.047). However, severe hypoglycaemia occurred less frequently with conventional control (RR (95% CI) = 0.22 (0.09–0.52), *P* = 0.001).

**Conclusions:**

This meta-analysis of intensive glycaemic control shows no association with reduced mortality in TBI. Intensive glucose control showed a borderline significant reduction in the risk of poor neurological outcome, but markedly increased the risk of hypoglycaemia. These contradictory findings should motivate further research.

**Electronic supplementary material:**

The online version of this article (10.1186/s13054-017-1883-y) contains supplementary material, which is available to authorized users.

## Background

Hyperglycaemia occurs frequently in the early phase following traumatic brain injury (TBI) and is associated with poor outcomes [1–3]. While marked acute hyperglycaemia appears to be toxic, the magnitude of the elevation in blood glucose required to cause harm remains uncertain. The pathogenesis of “stress hyperglycaemia” is broadly thought to represent a complex interplay between endogenous catecholamines, cytokines and activation of the hypothalamic-pituitary-adrenal axis resulting in excessive cortisol secretion and induction of gluconeogenesis [4]. This is further exacerbated by therapeutic interventions such as the administration of exogenous catecholamines and steroids, and any underlying insulin resistance or impaired insulin secretion [4]. Putative pathophysiological mechanisms by which hyperglycaemia may worsen TBI outcome include promotion of oxidative stress pathways and induction of neuroinflammation [5].

Current management of hyperglycaemia in TBI involves the use of short-acting insulin administered as a continuous intravenous infusion, titrated to maintain systemic blood glucose within target ranges that have been derived from randomised controlled trials in general medical or surgical intensive care unit (ICU) populations. The seminal single-centre study by Van den Berghe et al. resulted in a paradigm shift in the approach to blood glucose management in the critically ill, based on their finding that targeting intensive glycaemic control (4.4–6.1 mmol/L) reduced mortality in a surgical ICU population, even though there was an increased incidence of hypoglycaemia [6]. While there was considerable uptake of this regimen, subsequent multi-centre randomised controlled trials confirmed a higher incidence of severe hypoglycaemia with intensive glycaemic control [7–9] and refuted the initial observations by identifying a substantial increase in mortality with intensive blood glucose control [10].

It has been suggested that patients with TBI represent a unique subgroup of critically ill patients who have heightened susceptibility to both hyperglycaemia and hypoglycaemia [11, 12]. Accordingly, the results of randomised controlled trials in heterogeneous cohorts of critically ill patients may not be applicable to this population. The importance of considering patients with TBI as a distinct population is emphasized by the publication of subgroup analyses of patients with TBI from the aforementioned large randomised controlled trials [13, 14]. A comprehensive overview of blood glucose control focussed on the TBI population has never been performed and there are no contemporary guidelines for the optimal glycaemic range in this population [15]. Accordingly, we performed a systematic review and meta-analysis to determine whether intensive insulin therapy is associated with improved neurological outcomes and reduced mortality in TBI.

## Methods

### Protocol and registration

The protocol was written according to the preferred reporting items for systematic reviews and meta-analysis protocols (PRISMA)-P statement and was registered in the PROSPERO database (registration number CRD42016044071) [16–18]. The PRISMA-P and PRISMA checklists were used.

### Study eligibility criteria

Eligible studies were randomised controlled trials comparing intensive with conventional glycaemic control in adult patients (age > 16 years) with TBI requiring admission to a critical care unit. The cut-off for intensive glycaemic control was defined as those studies aiming for “normal values” (< 7.0 mmol/L) [19]. Conventional glucose control was defined as either moderate (upper limit of target range < 10 mmol/L) or liberal (upper limit of target range ≥ 10 mmol/L) in line with the “Normoglycemia in intensive care evaluation and surviving using glucose algorithm regulation” (NICE-SUGAR) trial [20]. Studies reported in any language were considered without publication date restriction. Paediatric studies, animal studies and observational, quasi-randomised or cross-over studies were excluded.

### Search strategy

We performed an unrestricted electronic database search in Medline (via Ovid®), Embase (via Ovid®) and the Cochrane Central Register of Controlled Trials (CENTRAL) from their inception date until 15 November 2016. Search details are provided in Additional file 1. After merging the searches in EndNote® (X7) and removing any duplicates, JH and MPP independently screened the titles and abstracts of all identified studies. Full texts of relevant studies were then evaluated for eligibility. Reference lists of retrieved papers were also reviewed to identify potentially eligible studies not captured in the primary search. If a sub-analysis was reported, the original paper from the main study was retrieved. Discrepancies between the investigators were discussed and, if unresolved, JPC and DKM were consulted. A translator was consulted to assist with papers published in a foreign language.

### Data collection

Extracted data included study characteristics (author, publication year, country, design, funding source and sample size), patient characteristics (demographics, diabetes mellitus status), intervention and comparator parameters (target range, duration of intervention, additional treatment), outcomes and results (definition of hypoglycaemia, definition of poor neurological outcome, definition of infective complications, follow-up time). If relevant data were not reported, the corresponding author was contacted and a response awaited for 2 weeks. Thereafter a reminder was sent and a reply awaited for a further 4 weeks.

### Bias assessment

Two reviewers (JH and MPP) independently and in duplicate assessed risk of bias among included studies using the Cochrane risk-of-bias tool [21]. The following domains were assessed: selection bias, performance bias, detection bias, attrition bias, reporting bias and other bias. A funnel plot was planned to assess bias if including > 10 studies.

### Outcome measures

Primary outcomes included:

1. ICU, in-hospital, 30-day and 90-day mortality (dichotomous)
2. 2, Poor neurological outcome as defined by the author (dichotomous)
3. Mild and severe hypoglycaemia as defined by the author (dichotomous)
4. Infective complications (dichotomous, i.e. wound infection, central nervous system (CNS) infection, pneumonia, sepsis/bacteraemia, cystitis/urine tract infection; defined using criteria used by the authors).

At least one of these outcomes had to be reported for inclusion in the final analysis.

### Data analysis

Primary analysis for the listed outcomes was performed as pairwise meta-analyses using the intensive and conventional groups as defined in the respective studies. Given the intrinsic differences in study design, outcome definitions and target glycaemic ranges employed, data were pooled and meta-analyses performed using random effects models, with effect estimates presented as relative risk (RR) and 95% confidence interval (95% CI). Heterogeneity was estimated by the *I*^2^-statistic. A secondary network meta-analysis was performed, based upon the target ranges intensive (< 7.0 mmol/L), moderate (< 10 mmol/L) and liberal (≥ 10 mmol/L); with the moderate and liberal groups compared by an adjusted indirect treatment comparison (AITC) method [22]. For consistency in direction of relative effects, the primary meta-analyses were performed with intensive control as the reference group. All analyses were performed in Stata MP 14.2.

## Results

### Study selection

**Fig. 1:**
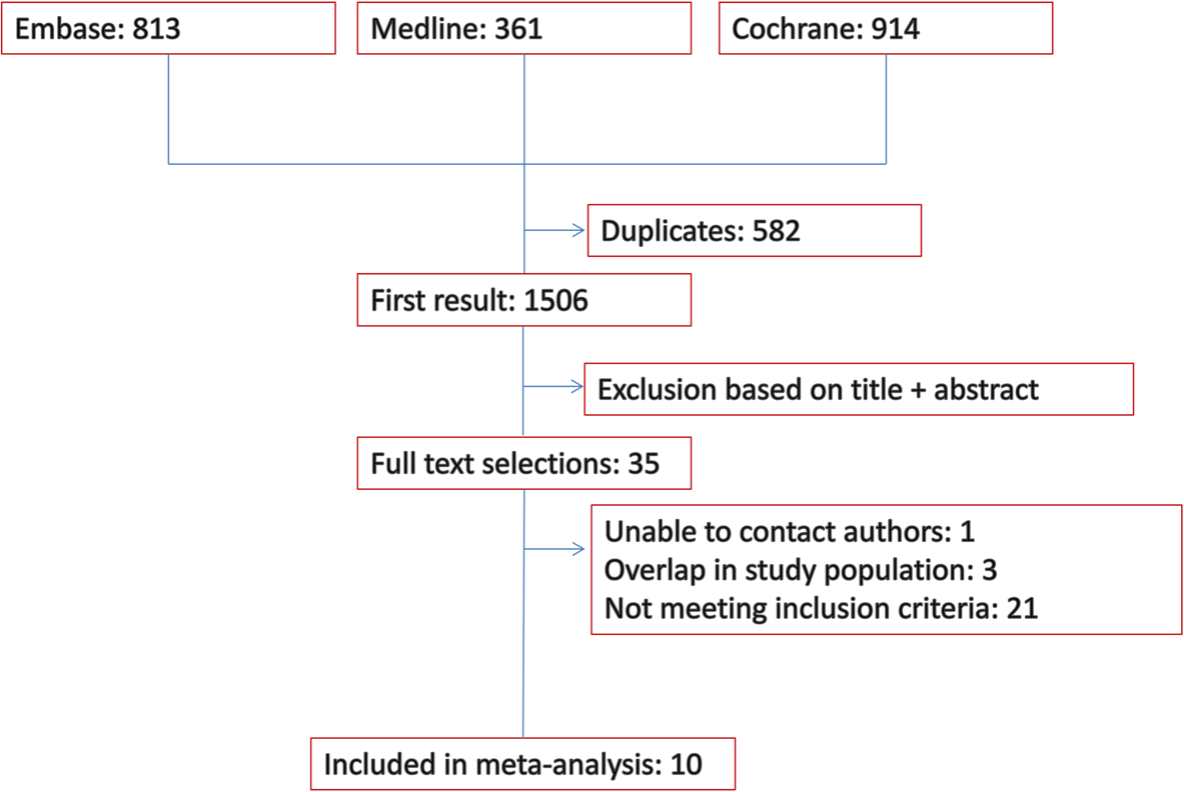
Flow chart of study selection

After removing duplicates, 1506 studies were evaluated for inclusion (Fig. 1). Following abstract and summary screening, 35 studies met the inclusion criteria for full-text extraction. A further 21 studies were excluded for not meeting the inclusion criteria or due to duplication (*n* = 3). In five of the included studies, it was clear after review of the full text that patients with TBI were included in the study population [7, 8, 14, 23, 24]. These authors were contacted and kindly provided data on the outcome variables of interest for the TBI sub-population (i.e. Arabi 2008 and 2011, Cinotti 2014, de la Rosa 2008, van den Berghe 2005). In total, ten studies were included in the final analysis (Table 1).

**Table 1:** Summary of included studies

Study ID^A^	Study description	Patients with TBI, *n*		IIT target	Control target	Moderate hypoglycaemia definition	Severe hypoglycaemia definition	Poor neurological outcome definition	Infectious complications	ICU mortality assessed?	Hospital mortality assessed?	30-Day mortality assessed?	90-Day mortality assessed?
		IIT	Control										
Arabi 2008 [7]	Single centre, medical/surgical ICU patients. IIT vs control (*n* = 523)	55	39	4.4–6.1 mmol/L	10–11.1 mmol/L	NA	≤2.2 mmol/L	NA	Occurring after 48 h of ICU admission and until 48 h after ICU discharge^b^	Yes	Yes	NA	NA
Arabi 2011 [24]	Single centre, medical/surgical ICU patients. IIT vs control (*n* = 240). 2x2 factorial design, other comparison permissive underfeeding vs target feeding	34	32	4.4–6.1 mmol/L	10–11.1 mmol/L	NA	≤2.2 mmol/L	NA	Occurring up to 48 h after ICU discharge^c^	Yes	Yes	NA	NA
Bilotta, 2008 [25]	Single centre, Patients with TBI admitted to ICU (*n* = 97), IIT vs control	48	49	4.4–6.7 mmol/L	<12 mmol/L	<4.44 mmol/L	NA	(GOS) at 6 months	Infections in the ICU^d^	NA	NA	NA	NA
Cinotti 2014 [23]	Sub-analysis of multi-centre study, 2 centres, brain injury requiring ICU admission, *n* = 188, computer-assisted IIT vs control	22	19	4.4–6.0 mmol/L	5.5–9 mmol/L	<3.3 mmol/L	<2.2 mmol/L	GOS 3–5 at 28 and 90 days	Pneumonia, 2005 American Thoracic Society (ATS) definition	Yes	NA	Yes	Yes
Coester 2010 [26]	Blunt TBI with ICU admission, single centre, *n* = 79	39	40	4.4–6.1 mmol/L	<10 mmol/L	<4.4 mmol/L	<2.2 mmol/L	GOS at 6 months	Sepsis and or pneumonia in the ICU^e^	Yes	Yes	NA	NA
De la Rosa 2008 [8]	Single centre medical/surgical ICU patients, IIT vs control, *n* = 504	46	44	4.4–6.1 mmol/L	10–11.1 mmol/L	2.2–3.2 mmol/L	<2.2 mmol/L	NA	ICU acquired infections^f^	Yes	Yes	Yes	NA
Green 2010 [28]	Single centre, mechanically ventilated neurologic patients in the ICU, IIT vs control, *n* = 81	22	18	4.4–6.1 mmol/L	<8.4 mmol/L	<3.3 mmol/L	<2.2 mmolL	mRS 3–5 at 90 days	Bloodstream infections and pneumonia in the ICU^g^	NA	NA	NA	Yes
NICE-SUGAR 2015 [13]	Sub-analysis of multi-centre trial surgical/medical ICUs, IIT vs control, *n* = 6104	166	149	4.5–6.0 mmol/L	<10 mmol/L	2.3–3.9 mmol/L	≤2.2 mmol/L	GOSE 1 − 4 at 24 months	Positive blood cultures during admission	Yes	Yes	NA	Yes
Van den Berghe 2005 [14]	Sub-analysis of single centre surgical ICU patients, IIT vs control, *n* = 1548	4	7	4.4–6.1 mmol/L	<12 mmol/L	<3.3 mmol/L	<2.2 mmol/L	Karnofsky score <60 at 12 months (deaths <60)	Positive blood cultures during admission	Yes	Yes	Yes	NA
Yang 2009 [27]	Single centre, TBI in neurocritical care patients, IIT vs control, *n* = 233	117	116	4.4–6.1 mmol/L	<11.2 mmol/L	NA	<2.2 mmol/L	GOS 1–3 at 6 months	ICU infection rate^h^	Yes	NA	NA	NA

*TBI* traumatic brain injury, *IIT* intensive insulin therapy, *GOS* Glasgow Outcome Scale, *GOSE* extended Glasgow Outcome Scale, *mRS* modified Rankin Scale

^a^First author, year published

^b^Sepsis, severe sepsis and septic shock were defined according to the 2001 International Sepsis Definitions Conference and types of nosocomial infections were defined according to the National Nosocomial Infections Surveillance System (NNIS)

^c^Including bacteremia, catheter-related bloodstream infection, urinary tract infection, ventilator-associated pneumonia and skin and soft tissue infections identified using the NNIS

^d^Wound infections, pneumonia, urinary infections, bacteremia, defined according to the NNIS

^e^Pneumonia (temperature 38.5 °C, white blood count, positive sputum culture and new infiltrate on chest radiograph); sepsis (documented or suspected infection plus two of four signs of systemic inflammation)

^f^CDC definition

^g^Bloodstream infections (growth on blood culture not from contaminant) and pneumonia (infiltrate on chest radiograph and growth on sputum culture). Blood cultures and sputum cultures when temperature >101 °F or at the discretion of the treating physician

^h^Pneumonia, sepsis and urinary and wound infections. Infection was defined according to the NNIS

### Study characteristics

**Fig. 2:**
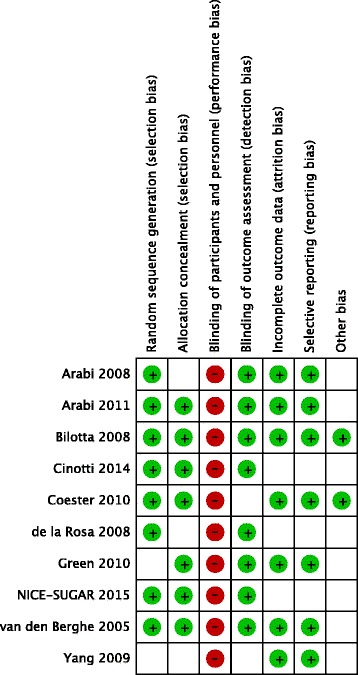
Risk of bias summary

All selected studies used a randomised trial to compare intensive with conventional glycaemic control in patients with TBI admitted to a (neuro) ICU (Table 1). Only the studies from Bilotta 2008, Coester 2010 and Yang 2009 were performed exclusively in patients with TBI [25–27]. In the study by Green 2010, data on patients with TBI were published as a subset of neurological patients admitted to an ICU [28]. The NICE-SUGAR investigators (NICE-SUGAR 2015) published a subgroup analysis of patients with TBI [10] and the study from van den Berghe 2005 is an analysis of neurological patients from the 2001 Leuven trial [6]. Cinotti 2014 is a subgroup analysis of patients with TBI from two centres included in the CGAO-REA study [29]. Arabi 2008 and 2011 and de la Rosa 2008, were performed in single-centre mixed medical/surgical ICUs [7, 8, 24]. Nine out of 10 studies had an intensive target range of 4.4–6.0 (or 6.1) mmol/L [7, 8, 13, 14, 23, 24, 26–28]. Only the study by Bilotta 2008 aimed for 4.4–6.7 mmol/L in the intensive arm [25]. The target range for conventional glycaemic control was more variable, with one study aiming for < 12 mmol/L [25], five studies for 10–11.1 mmol/L [7, 8, 14, 24, 27], two studies for < 10 mmol/L [13, 26], one for 5.5–9.0 mmol/L [23] and one study aiming for < 8.4 mmol/L [28]. The summary of the assessment of risk of bias is displayed in Fig. 2. Inherent to studies on intensive glycaemic control, none are double-blinded. Three studies did not provide details on allocation concealment. Blinding of outcome assessment was only considered relevant when neurological outcome was assessed. For two studies it was unclear whether this was performed in a blinded fashion [26, 30]. One study used a factorial design but there was no suggestion of interaction and hence the outcomes were included in the final analysis [24]. Subgroup analyses for patients with and without diabetes mellitus were not available. Most studies did not exclusively study patients with TBI, and we scored other bias as “unclear” because the subgroup analyses may bring about bias.

### Mortality

**Fig. 3:**
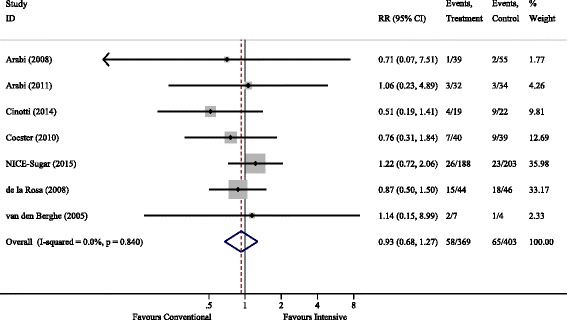
ICU mortality. RR, relative risk

ICU and hospital mortality data were available in seven studies. There was no difference in mortality between conventional and intensive glycaemic control, with the relative risk (RR) (95% CI) being 0.93 (0.68 − 1.27), *P* = 0.64 and 1.07 (0.84–1.355), *P* = 0.62 for ICU and hospital mortality respectively. Heterogeneity was non-significant. Both 30-day and 90-day mortality were reported in three studies each, with the RR (95% CI) being 0.78 (0.47 − 1.30) and 0.90 (0.50–1.65) respectively. The forest plot for ICU mortality is shown in Fig. 3.

Secondary network meta-analysis showed a non-significant lower risk for both liberal and conventional control compared with tight control, with RR (95% CI) 0.90 (0.55–1.46) and 0.91 (0.55–1.48), respectively, for ICU mortality and with 0.99 (0.74–1.33) and 1.24 (0.83 − 1.86) for hospital mortality. The indirect estimates for liberal versus moderate control were 0.99 (0.49–1.98) and 0.80 (0.49–1.32) for ICU and hospital mortality, respectively.

### Neurological outcome

**Fig. 4:**
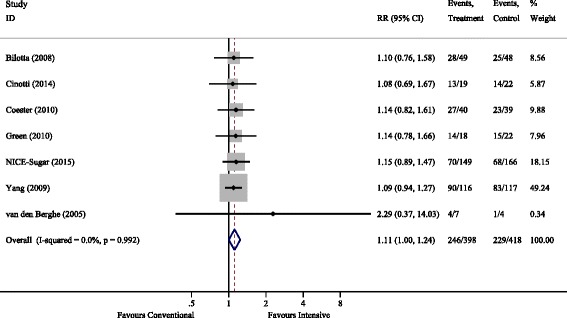
Poor neurological outcome. RR, relative risk

Seven studies provided data on neurological outcome albeit at different time points. Cinotti 2014 had the earliest measurement at 28 days, which was repeated at three months [23]. For the analysis we used an outcome of ≥90 days, as all other studies used a similar or later time point. Thus the analysed time range is 3–24 months. Most studies used the Glasgow Outcome Scale (GOS) [23, 26, 27], or the extended Glasgow Outcome Scale (GOSE) [13]. The general definition of poor neurological outcome was a GOS of 1–3 or GOSE of 1–4 (ranging from severe disability to death) [31]. Only Cinotti 2014 defined poor neurological outcome as vegetative state or death [23]. When the authors provided data for all GOS scores, we used GOS 1–3 as the definition of poor neurological outcome. Green 2010 used the modified Rankin scale (mRS) and defined poor outcome as a score > 2 (moderate disability-death) [28]. Finally, Van den Berghe 2006 used the Karnofsky score, defining poor neurological outcome as < 60 (> 60 indicates one is able to care for most needs) [14]. All of the poor neurological outcome scores include death. The pooled RR (95% CI) for poor neurological outcome for conventional versus intensive control was 1.11 (1.001–1.239), *P* = 0.047. The forest plot for poor neurological outcome is shown in Fig. 4.

Network meta-analysis showed a non-significant increase in risk of an unfavourable outcome for both liberal and moderate control compared with tight control, with RR (95% CI) = 1.10 (0.96–1.26) and 1.14 (0.96–1.34), respectively and with the indirect estimate for liberal versus moderate control = 0.97 (0.78–1.20), which was also non-significant.

### Hypoglycaemia

Nine studies defined severe hypoglycaemia as blood glucose < 2.2 or ≤ 2.2 mmol/L except for Bilotta, who did not report severe hypoglycaemia [25]. Five studies reported moderate hypoglycaemia which was variably defined as blood glucose of 2.2–3.2 to < 4.44 mmol/L [13, 14, 25, 26, 28]. We included patients with one or more episodes of hypoglycaemia.

**Fig. 5:**
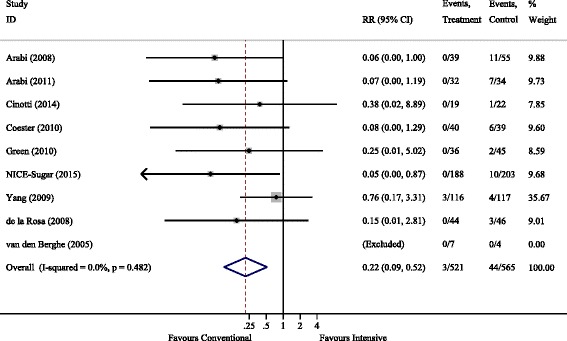
Severe hypoglycaemic events. RR, relative risk

**Fig. 6:**
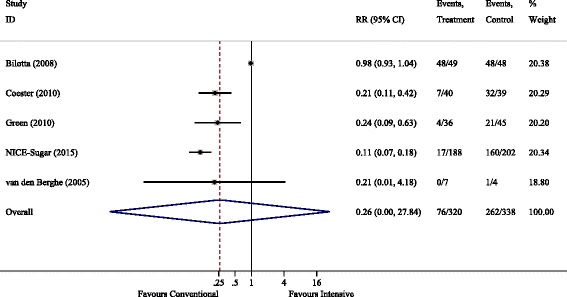
Moderate hypoglycaemic events. RR, relative risk

There was a clear association between intensive glycaemic control and severe hypoglycaemia with the RR (95% CI) for conventional control of 0.22 (0.09–0.52), *P* = 0.001. For moderate hypoglycaemia there was marked heterogeneity between studies, with *I*^2^ > 95%, and RR = 0.26 (0.00–27.8) being non-significant. Forest plots are shown in Figs. 5 and 6.

Network meta-analysis also showed a lower risk of hypoglycaemia for both the liberal and moderate control compared with tight control. For severe hypoglycaemia the liberal RR (95% CI) was 0.30 (0.10–0.87), *P* = 0.03 and moderate 0.13 (0.03–0.57), *P* = 0.007, respectively, with the indirect estimate for liberal versus moderate control being 2.28 (0.37–14.2), *P* = 0.38. For moderate hypoglycaemia the RR for liberal control was 0.92 (0.49–1.71), *P* = 0.78 and for moderate control it was 0.16 (0.10–0.26), *P* < 0.001, with an indirect estimate for liberal versus moderate control of 5.7 (2.57–12.6), *P* < 0.001.

### Infective complications

The definitions of infective complications are depicted in Table 1. The marked heterogeneity in defining infective complications precluded formal meta-analysis. Most studies did not find a significant difference in infective complications. The two exceptions were the study by de la Rosa 2008 in which there was a reduction in thea combined endpoint of ICU-acquired ventilator-associated pneumonia, bloodstream infections and urinary tract infections in the intensive-glycaemic-control group (RR 0.24, 95% CI 0.08–0.68) [8] and the study by Yang 2009 who reported a reduction in infective complications (pneumonia, sepsis, urinary tract and wound infections) by 14.8% in the intensive-glycaemic-control group, *P* < 0.05 [27].

## Discussion

We undertook the first meta-analysis of randomised controlled trials comparing conventional with intensive glycaemic control in critically ill patients with TBI. Previous systematic reviews in a neurocritical care setting have been published; however, these have failed to distinguish patients with TBI from those with non-traumatic brain injury (stroke, encephalitis and central nervous system (CNS) infections) [32, 33] and did not include relevant recent publications [13, 23]. In addition, we were able to include unpublished data from relevant trials that had previously not been included in systematic reviews in this setting [7, 8, 14, 23, 24].

We report that intensive glycaemic control shows no association with reduced mortality in patients with TBI. This observation is consistent with results from the broader neurocritical care population [32, 33] and multi-centre studies of heterogeneous mixed medical/surgical populations [9, 10, 29, 34]. The observed signal, that intensive glycaemic control may be associated with a lower risk of poor neurological outcome, is consistent with the findings by Kramer et al. in their meta-analysis of glycaemic control among all neurocritical care admissions [32]. In contrast, severe hypoglycaemia was markedly increased with intensive glycaemic control. Due to heterogeneity in classification, there was insufficient evidence to determine the effect of glycaemic control strategies on the incidence of infective complications.

The lower risk of poor neurological outcome with intensive glycaemic control was remarkably consistent across all included studies, as shown in Fig. 4. This consistency is surprising, especially given the different study populations and different outcome metrics employed, i.e. neurological outcome being analysed as a composite of multiple scoring systems across a broad range of follow-up periods from 3 to 24 months. Accordingly, we believe this finding should be interpreted with some caution; however, it should certainly be viewed as hypothesis-generating. Potential mechanisms for the harmful effect of conventional glucose targets or a beneficial effect of intensive control include, amongst others, hyperglycaemia-induced mitochondrial dysfunction [35] and oxidative stress [4, 36]. In the Van den Berghe 2005 study, intensive glucose control was associated with lower intracranial pressure, fewer seizures and a lesser incidence of diabetes insipidus [14]. There were only eleven patients with TBI in this sub-study and no other studies in this analysis reported these outcome measures. Subsequent meta-analyses would benefit from future trials in this area reporting a core set of neurological outcome measures [37].

We report a strong association between intensive glycaemic control and hypoglycaemia and there is mechanistic plausibility that hypoglycaemia causes harm. Hypoglycaemia has a strong dose-dependent association with mortality in critically ill patients [38, 39] and aggravates critical illness neurocognitive dysfunction [40]. The brain is unique in that it has high demands for energy and, due to a limited capacity to store glycogen, is vulnerable to periods of reduced substrate provision [12]. Moreover, cerebral glucose metabolism is altered in a non-uniform way following TBI [41]. Although global glucose metabolism is decreased, areas of increased activity are also observed [42]. Accordingly, lowering glucose in patients with TBI might predispose areas of hyper-metabolism to glucose deprivation. This hypothesis is supported by microdialysis studies of cerebral glucose metabolism demonstrating that intensive glycaemic control is associated with neuroglycopenia and surrogate markers of brain energy crisis in the absence of systemic hypoglycaemia [43, 44]. The finding from the network meta-analysis that there is an increased risk of hypoglycaemia with liberal control as compared to the moderate control is unexpected and should be interpreted with caution as this may relate to differences in the underlying studies, and is in contradistinction to the recent comprehensive network meta-analysis of glucose control in adult critically ill patients from Yamada et al. [45]. This systematic review and network meta-analysis of 36 randomised controlled trials included all nine studies reporting hypoglycaemia in TBI population from the present analysis, and demonstrated a stepwise increase in hypoglycaemia with tight control (glucose 4.4 to < 6.1 mmol/L) compared to mild control (7.8 to < 10.0 mmol/L) and very mild control (10.0 to 12.2 mmol/L) [45].

Strengths of our meta-analysis include the structured search; the specificity of the population studied; the inclusion of publications in any language and unpublished data; the validated methods in accordance with the PRISMA-P statement and the secondary network analysis for indirect between-group effects. However, our study has several important limitations. The heterogeneity in glycaemic targets may potentially obscure any between-group outcome effects. In order to address this concern we undertook a secondary network analysis to identify differences between levels of conventional control (Additional file 1: Figure S1); unfortunately, given small patient numbers this analysis was likely underpowered for the listed outcomes, increasing the risk of the influence of random error on bias estimates, which may explain the differing results from the Yamada review. The aforementioned heterogeneity in the timing and classification of “poor neurological outcome” weakens any inference that might otherwise be made, despite the lack of statistical heterogeneity observed. Severe hypoglycaemic events occurred with zero count in the conventional arm in eight of the nine studies included; the standard software application of a continuity correction of 0.5 to each cell in such a large proportion of studies may bias the effect estimate [46]; however, the direction and significance of this effect persisted when analysing the studies as rate differences, with the continuity correction set to zero – see Additional file 1: Figure S2.

Inherent to randomised controlled trials of glucose control in the critically ill, there was no blinding of patient or physician to treatment arm, such that all studies carried a high risk of bias in this domain. Furthermore, between-trial differences in monitoring of blood glucose with regard to sampling frequency and measurement method are a source of heterogeneity [47]. In addition, the neurological outcome was assessed after 3–24 months, whereas one could argue that assessment within 1 year after TBI may be too early [48]. Finally, only three of the trials exclusively studied TBI; in the other trials this was addressed in a subgroup analysis. Although some studies used stratification for admission diagnosis or medical speciality, this brings about an unclear risk of bias. If one would design an adequately powered trial based on neurological outcome, the sample size would be > 775 patients, at least, per group.

We did not achieve complete retrieval of all identified studies. The GLUCONTROL trial authors kindly provided available data; however, patients with TBI were not a defined subgroup within the neurological diagnostic category (*n* = 148) [9] such that these data were excluded. There were incomplete data on the outcomes of interest, with mortality and neurological outcome data only available for 7 of the 10 included studies increasing the risk of random error influencing bias estimates and decreasing the strength of the inferences that can be made. Finally, while nine of the studies identified patients with diabetes mellitus, data on the outcomes for this subgroup were not available. The incidence of diabetes among patients with TBI is estimated at 3% [49]. This is likely to be important, as evidence from the general intensive care population suggests that chronic blood glucose control modifies the associations between acute glycaemia and outcome in critical illness [50–53]. Future prospective studies in this area would benefit from the presentation of diabetic performance as a pre-defined subgroup of interest.

### Recommendations and future directions

Both hypoglycaemia and hyperglycaemia may be harmful in patients with TBI. These data show that regimens of glucose control aiming for intensive glycaemic targets (plasma glucose < 7.0 mmol/L) with short-acting intravenous insulin infusions, adjusted according to paper-based or computer algorithms, targeting population-based blood sugar targets, may improve neurological outcome, but at the risk of a relatively high incidence of severe hypoglycaemia. Given the limitations of this meta-analysis and these contradictory findings, we believe there is insufficient evidence to treat patients with TBI differently from other critically ill patients and we advocate a more moderate approach with a target < 10 mmol/L as recommended by NICE-SUGAR [13]. With the advent of reliable “closed-loop” insulin administration and adjunct glucose-lowering agents with a low risk of hypoglycaemia, such as glucagon-like peptide-1 (GLP-1)-based therapies [54], the risk of hypoglycaemia may decrease and the benefits of strict control may need to be reassessed, particularly given the signal for improved neurological outcomes with this strategy.

We also need to consider emerging data suggesting that the relationship between brain and blood glucose levels varies substantially between patients (and perhaps at different time points following injury) [41, 55]. A single “one size fits all” blood glucose target may be suboptimal in such a heterogeneous patient population, but titration to the needs of individual patients will only be possible if we can monitor brain glucose, possibly using microdialysis. While this monitoring modality is still not widely available, current large studies are accumulating data in this regard [56], and may be able to define subsets of patients with TBI for stratified blood glucose targets using comparative effectiveness research (CER) approaches.

We suggest therefore, that further investigation of this issue adopts a phased approach. Initial studies will need to refine safer approaches to intensive blood sugar control (with closed loop and GLP-1-based approaches), while the identification of patient groups for stratified blood glucose targets evolves from CER analyses of ongoing studies. Eventually, the combination of safer intensive glycaemic control and patient-specific blood glucose targets could then be evaluated in a definitive randomized controlled trial (RCT) with adequate power and sufficient sample size.

## Conclusions

Intensive glycaemic control does not reduce mortality in patients with TBI but greatly increases the risk of hypoglycaemia. A signal toward improved neurological outcome with intensive glycaemic control in patients post TBI warrants investigation. This may be best undertaken using safer approaches to glycaemic control that reduce the risk of hypoglycaemia, using stratified blood sugar targets that take account of physiological heterogeneity in patients with TBI.

## Additional file

Additional file 1: Search strategy and network meta-analysis figures. (DOC 117 kb)

## Abbreviations

CER: Comparative effectiveness research
CI: Confidence interval
CNS: Central nervous system
GLP-1: Glucagon-like peptide-1
GOS: Glasgow Outcome Scale
GOSE: Extended Glasgow Outcome Scale
ICU: Intensive care unit
mRS: Modified Rankin Scale
RCT: Randomised controlled trial
RR: Relative risk
TBI: Traumatic brain injury

## References

[1] Jeremitsky E, Omert LA, Dunham CM, Wilberger J, Rodriguez A (2005). The impact of hyperglycemia on patients with severe brain injury. J Trauma.

[2] Young B, Ott L, Dempsey R, Haack D, Tibbs P (1989). Relationship between admission hyperglycemia and neurologic outcome of severely brain-injured patients. Ann Surg.

[3] Griesdale DE, Tremblay MH, McEwen J, Chittock DR (2009). Glucose control and mortality in patients with severe traumatic brain injury. Neurocrit Care.

[4] Dungan KM, Braithwaite SS, Preiser JC (2009). Stress hyperglycaemia. Lancet.

[5] Jauch-Chara K, Oltmanns KM (2014). Glycemic control after brain injury: boon and bane for the brain. Neuroscience.

[6] van den Berghe G, Wouters P, Weekers F, Verwaest C, Bruyninckx F, Schetz M, Vlasselaers D, Ferdinande P, Lauwers P, Bouillon R (2001). Intensive insulin therapy in critically ill patients. N Engl J Med.

[7] Arabi YM, Dabbagh OC, Tamim HM, Al-Shimemeri AA, Memish ZA, Haddad SH, Syed SJ, Giridhar HR, Rishu AH, Al-Daker MO (2008). Intensive versus conventional insulin therapy: a randomized controlled trial in medical and surgical critically ill patients. Crit Care Med.

[8] De La Rosa GC, Donado JH, Restrepo AH, Quintero AM, Gonzalez LG, Saldarriaga NE, Bedoya M, Toro JM, Velasquez JB, Valencia JC (2008). Strict glycaemic control in patients hospitalised in a mixed medical and surgical intensive care unit: a randomised clinical trial. Crit Care.

[9] Preiser JC, Devos P, Ruiz-Santana S, Melot C, Annane D, Groeneveld J, Iapichino G, Leverve X, Nitenberg G, Singer P (2009). A prospective randomised multi-centre controlled trial on tight glucose control by intensive insulin therapy in adult intensive care units: the Glucontrol study. Intensive Care Med.

[10] Investigators N-SS, Finfer S, Chittock DR, Su SY, Blair D, Foster D, Dhingra V, Bellomo R, Cook D, Dodek P (2009). Intensive versus conventional glucose control in critically ill patients. N Engl J Med.

[11] Godoy DA, Di Napoli M, Rabinstein AA (2010). Treating hyperglycemia in neurocritical patients: benefits and perils. Neurocrit Care.

[12] Jalloh I, Carpenter KL, Helmy A, Carpenter TA, Menon DK, Hutchinson PJ (2015). Glucose metabolism following human traumatic brain injury: methods of assessment and pathophysiological findings. Metab Brain Dis.

[13] Blair D, Norton R, Dhingra V, Foster D, Hebert P, Henderson W, Heyland D, Ronco J, Peto R, Sandercock P (2015). Intensive versus conventional glucose control in critically ill patients with traumatic brain injury: long-term follow-up of a subgroup of patients from the NICE-SUGAR study. Intensive Care Med.

[14] Van den Berghe G, Schoonheydt K, Becx P, Bruyninckx F, Wouters PJ (2005). Insulin therapy protects the central and peripheral nervous system of intensive care patients. Neurology.

[15] Carney N, Totten AM, O’Reilly C, Ullman JS, Hawryluk GW, Bell MJ, Bratton SL, Chesnut R, Harris OA, Kissoon N, Rubiano AM, Shutter L, Tasker RC, Vavilala MS, Wilberger J, Wright DW, Ghajar J (2017). Guidelines for the management of severe traumatic brain injury, Fourth Edition. Neurosurg..

[16] Moher D, Shamseer L, Clarke M, Ghersi D, Liberati A, Petticrew M, Shekelle P, Stewart LA (2015). Preferred reporting items for systematic review and meta-analysis protocols (PRISMA-P) 2015 statement. Syst Rev.

[17] Straus S, Moher D (2010). Registering systematic reviews. CMAJ.

[18] Liberati A, Altman DG, Tetzlaff J, Mulrow C, Gøtzsche PC, Ioannidis JP, Clarke M, Devereaux PJ, Kleijnen J, Moher D. The PRISMA statement for reporting systematic reviews and meta-analyses of studies that evaluate healthcare interventions: explanation and elaboration. BMJ. 2009:339. 10.1136/bmj.b2700 PMC2714672 19622552

[19] Association AD (2016). 2. Classification and diagnosis of diabetes. Diabetes Care.

[20] Investigators TN-SS (2009). Intensive versus conventional glucose control in critically ill patients. N Engl J Med.

[21] Higgins JPT, Altman DG, Gøtzsche PC, Jüni P, Moher D, Oxman AD, Savović J, Schulz KF, Weeks L, Sterne JAC (2011). The Cochrane Collaboration’s tool for assessing risk of bias in randomised trials. BMJ.

[22] Miladinovic B, Chaimani A, Hozo I, Djulbegovic B (2014). Indirect treatment comparison. Stata J.

[23] Cinotti R, Ichai C, Orban JC, Kalfon P, Feuillet F, Roquilly A, Riou B, Blanloeil Y, Asehnoune K, Rozec B (2014). Effects of tight computerized glucose control on neurological outcome in severe brain-injured patients. A multi-center sub-group analysis of the randomized-controlled open-label CGAO-REA study. Intensive Care Med.

[24] Arabi YM, Tamim HM, Dhar GS, Al-Dawood A, Al-Sultan M, Sakkijha MH, Kahoul SH, Brits R (2011). Permissive underfeeding and intensive insulin therapy in critically ill patients: a randomized controlled trial. Am J Clin Nutr.

[25] Bilotta F, Caramia R, Cernak I, Paoloni FP, Doronzio A, Cuzzone V, Santoro A, Rosa G (2008). Intensive insulin therapy after severe traumatic brain injury: a randomized clinical trial. Neurocrit Care.

[26] Coester A, Neumann CR, Schmidt MI (2010). Intensive insulin therapy in severe traumatic brain injury: a randomized trial. J Trauma.

[27] Yang M, Guo Q, Zhang X, Sun S, Wang Y, Zhao L, Hu E, Li C (2009). Intensive insulin therapy on infection rate, days in NICU, in-hospital mortality and neurological outcome in severe traumatic brain injury patients: a randomized controlled trial. Int J Nurs Stud.

[28] Green DM, O’Phelan KH, Bassin SL, Chang CW, Stern TS, Asai SM (2010). Intensive versus conventional insulin therapy in critically ill neurologic patients. Neurocrit Care.

[29] Kalfon P, Giraudeau B, Ichai C, Guerrini A, Brechot N, Cinotti R, Dequin PF, Riu-Poulenc B, Montravers P, Annane D (2014). Tight computerized versus conventional glucose control in the ICU: a randomized controlled trial. Intensive Care Med.

[30] Van Den Berghe G, Wilmer A, Milants I, Wouters PJ, Bouckaert B, Bruyninckx F, Bouillon R, Schetz M (2006). Intensive insulin therapy in mixed medical/surgical intensive care units: benefit versus harm. Diabetes.

[31] Teasdale GM, Pettigrew LE, Wilson JT, Murray G, Jennett B (1998). Analyzing outcome of treatment of severe head injury: a review and update on advancing the use of the Glasgow Outcome Scale. J Neurotrauma.

[32] Kramer AH, Roberts DJ, Zygun DA (2012). Optimal glycemic control in neurocritical care patients: a systematic review and meta-analysis. Crit Care.

[33] Zafar SN, Iqbal A, Farez MF, Kamatkar S, de Moya MA (2011). Intensive insulin therapy in brain injury: a meta-analysis. J Neurotrauma.

[34] Brunkhorst FM, Engel C, Bloos F, Meier-Hellmann A, Ragaller M, Weiler N, Moerer O, Gruendling M, Oppert M, Grond S (2008). Intensive insulin therapy and pentastarch resuscitation in severe sepsis. N Engl J Med.

[35] Van den Berghe G, Schoonheydt K, Becx P, Bruyninckx F, Wouters PJ. Insulin therapy protects the central and peripheral nervous system of intensive care patients. Neurology. 2005;64(8):1348-53. 10.1212/01.WNL.0000158442.08857.FC 15851721

[36] Sonneville R, den Hertog HM, Guiza F, Gunst J, Derese I, Wouters PJ, Brouland JP, Polito A, Gray F, Chretien F (2012). Impact of hyperglycemia on neuropathological alterations during critical illness. J Clin Endocrinol Metab.

[37] Hodgson CL, Turnbull AE, Iwashyna TJ, Parker A, Davis W, Bingham CO, Watts NR, Finfer S, Needham DM (2017). Core domains in evaluating patient outcomes after acute respiratory failure: international multidisciplinary clinician consultation. Phys Ther.

[38] Investigators N-SS, Finfer S, Liu B, Chittock DR, Norton R, Myburgh JA, McArthur C, Mitchell I, Foster D, Dhingra V (2012). Hypoglycemia and risk of death in critically ill patients. N Engl J Med.

[39] Hermanides J, Bosman RJ, Vriesendorp TM, Dotsch R, Rosendaal FR, Zandstra DF, Hoekstra JB, DeVries JH (2010). Hypoglycemia is associated with intensive care unit mortality. Crit Care Med.

[40] Duning T, van den Heuvel I, Dickmann A, Volkert T, Wempe C, Reinholz J, Lohmann H, Freise H, Ellger B (2010). Hypoglycemia aggravates critical illness-induced neurocognitive dysfunction. Diabetes Care.

[41] Abate MG, Trivedi M, Fryer TD, Smielewski P, Chatfield DA, Williams GB, Aigbirhio F, Carpenter TA, Pickard JD, Menon DK (2008). Early derangements in oxygen and glucose metabolism following head injury: the ischemic penumbra and pathophysiological heterogeneity. Neurocrit Care.

[42] Hattori N, Huang SC, Wu HM, Liao W, Glenn TC, Vespa PM, Phelps ME, Hovda DA, Bergsneider M (2004). Acute changes in regional cerebral (18)F-FDG kinetics in patients with traumatic brain injury. J Nucl Med.

[43] Vespa P, Boonyaputthikul R, McArthur DL, Miller C, Etchepare M, Bergsneider M, Glenn T, Martin N, Hovda D (2006). Intensive insulin therapy reduces microdialysis glucose values without altering glucose utilization or improving the lactate/pyruvate ratio after traumatic brain injury. Crit Care Med.

[44] Oddo M, Schmidt JM, Carrera E, Badjatia N, Connolly ES, Presciutti M, Ostapkovich ND, Levine JM, Le Roux P, Mayer SA (2008). Impact of tight glycemic control on cerebral glucose metabolism after severe brain injury: a microdialysis study. Crit Care Med.

[45] Yamada T, Shojima N, Noma H, Yamauchi T, Kadowaki T (2017). Glycemic control, mortality, and hypoglycemia in critically ill patients: a systematic review and network meta-analysis of randomized controlled trials. Intensive Care Med.

[46] Sweeting MJ, Sutton AJ, Lambert PC (2004). What to add to nothing? Use and avoidance of continuity corrections in meta-analysis of sparse data. Stat Med.

[47] Krinsley JS, Bruns DE, Boyd JC (2015). The impact of measurement frequency on the domains of glycemic control in the critically ill–a Monte Carlo simulation. J Diabetes Sci Technol.

[48] Sandhaug M, Andelic N, Langhammer B, Mygland A (2015). Functional level during the first 2 years after moderate and severe traumatic brain injury. Brain Inj.

[49] Ley EJ, Srour MK, Clond MA, Barnajian M, Tillou A, Mirocha J, Salim A (2011). Diabetic patients with traumatic brain injury: insulin deficiency is associated with increased mortality. J Trauma.

[50] Plummer MP, Bellomo R, Cousins CE, Annink CE, Sundararajan K, Reddi BA, Raj JP, Chapman MJ, Horowitz M, Deane AM (2014). Dysglycaemia in the critically ill and the interaction of chronic and acute glycaemia with mortality. Intensive Care Med.

[51] Egi M, Bellomo R, Stachowski E, French CJ, Hart GK, Hegarty C, Bailey M (2008). Blood glucose concentration and outcome of critical illness: the impact of diabetes. Crit Care Med.

[52] Egi M, Bellomo R, Stachowski E, French CJ, Hart GK, Taori G, Hegarty C, Bailey M (2011). The interaction of chronic and acute glycemia with mortality in critically ill patients with diabetes. Crit Care Med.

[53] Sechterberger MK, Bosman RJ, Oudemans-van Straaten HM, Siegelaar SE, Hermanides J, Hoekstra JB, De Vries JH (2013). The effect of diabetes mellitus on the association between measures of glycaemic control and ICU mortality: a retrospective cohort study. Crit Care.

[54] Plummer MP, Chapman MJ, Horowitz M, Deane AM (2014). Incretins and the intensivist: what are they and what does an intensivist need to know about them?. Crit Care.

[55] O’Connell MT, Seal A, Nortje J, Al-Rawi PG, Coles JP, Fryer TD, Menon DK, Pickard JD, Hutchinson PJ (2005). Glucose metabolism in traumatic brain injury: a combined microdialysis and [18 F]-2-fluoro-2-deoxy-D-glucose-positron emission tomography (FDG-PET) study. Acta Neurochir Suppl.

[56] Maas AI, Menon DK, Steyerberg EW, Citerio G, Lecky F, Manley GT, Hill S, Legrand V, Sorgner A, Participants C-T (2015). Collaborative European NeuroTrauma Effectiveness Research in Traumatic Brain Injury (CENTER-TBI): a prospective longitudinal observational study. Neurosurgery.

