# Supplementary material for: Glycaemic control targets after traumatic brain injury: a systematic review and meta-analysis
Source: Crit Care. 2018 Jan 19;22:11. doi: 10.1186/s13054-017-1883-y (PMC5775599; doi:10.1186/s13054-017-1883-y)
Supplement: Additional file 1: — Search strategy and network meta-analysis figures. (DOC 117 kb) [file 13054_2017_1883_MOESM1_ESM.doc]

Additional file 1

Search strategy

Embase

| 1. | Clinical trial/ |  |
| --- | --- | --- |
| 2. | Randomized controlled trial/ |  |
| 3. | Randomization/ |  |
| 4. | Single blind procedure/ |  |
| 5. | Double blind procedure/ |  |
| 6. | Crossover procedure/ |  |
| 7. | Placebo/ |  |
| 8. | Randomi?ed controlled trial$.tw. |  |
| 9. | Rct.tw. |  |
| 10. | Random allocation.tw. |  |
| 11. | Randomly allocated.tw. |  |
| 12. | Allocated randomly.tw. |  |
| 13. | (allocated adj2 random).tw. |  |
| 14. | Single blind$.tw. |  |
| 15. | Double blind$.tw. |  |
| 16. | ((treble or triple) adj blind$).tw. |  |
| 17. | Placebo$.tw. |  |
| 18. | Prospective study/ |  |
| 19. | or/1-18 |  |
| 20. | Case study/ |  |
| 21. | Case report.tw. |  |
| 22. | Abstract report/ or letter/ |  |
| 23. | or/20-22 |  |
| 24. | 19 not 23 |  |
| 25. | traumatic brain injur*.mp. [mp=title, abstract, heading word, drug trade name, original title, device manufacturer, drug manufacturer, device trade name, keyword] |  |
| 26. | acute brain injur*.mp. [mp=title, abstract, heading word, drug trade name, original title, device manufacturer, drug manufacturer, device trade name, keyword] |  |
| 27. | brain injur*.mp. [mp=title, abstract, heading word, drug trade name, original title, device manufacturer, drug manufacturer, device trade name, keyword] |  |
| 28. | TBI.mp. [mp=title, abstract, heading word, drug trade name, original title, device manufacturer, drug manufacturer, device trade name, keyword] |  |
| 29. | neurological injur*.mp. [mp=title, abstract, heading word, drug trade name, original title, device manufacturer, drug manufacturer, device trade name, keyword] |  |
| 30. | brain injury/ |  |
| 31. | or/25-30 |  |
| 32. | intensive insulin therapy.mp. [mp=title, abstract, heading word, drug trade name, original title, device manufacturer, drug manufacturer, device trade name, keyword] |  |
| 33. | insulin/ |  |
| 34. | glucose/ |  |
| 35. | antidiabetic agent/ |  |
| 36. | intensive insulin.mp. [mp=title, abstract, heading word, drug trade name, original title, device manufacturer, drug manufacturer, device trade name, keyword] |  |
| 37. | 32 or 33 or 34 or 35 or 36 |  |
| 38. | 24 and 31 and 37 |  |
| 39. | ICU.mp. [mp=title, abstract, heading word, drug trade name, original title, device manufacturer, drug manufacturer, device trade name, keyword] |  |
| 40. | Intensive care.mp. [mp=title, abstract, heading word, drug trade name, original title, device manufacturer, drug manufacturer, device trade name, keyword] |  |
| 41. | Critically ill.mp. [mp=title, abstract, heading word, drug trade name, original title, device manufacturer, drug manufacturer, device trade name, keyword] |  |
| 42. | Critical Care.mp. [mp=title, abstract, heading word, drug trade name, original title, device manufacturer, drug manufacturer, device trade name, keyword] |  |
| 43. | intensive care/ or intensive care unit/ |  |
| 44. | 39 or 40 or 41 or 42 or 43 |  |
| 45. | 31 or 44 |  |
| 46. | 24 and 37 and 45 |  |
| 47. | limit 46 to (human and (adult <18 to 64 years> or aged <65+ years>)) |  |

Medline

| 1. | Randomized Controlled Trials as Topic/ |  |
| --- | --- | --- |
| 2. | randomized controlled trial/ |  |
| 3. | Random Allocation/ |  |
| 4. | Double Blind Method/ |  |
| 5. | clinical trial/ |  |
| 6. | clinical trial, phase i.pt. |  |
| 7. | clinical trial, phase ii.pt. |  |
| 8. | clinical trial, phase iii.pt. |  |
| 9. | clinical trial, phase iv.pt. |  |
| 10. | controlled clinical trial.pt. |  |
| 11. | randomized controlled trial.pt. |  |
| 12. | multicenter study.pt. |  |
| 13. | clinical trial.pt. |  |
| 14. | exp Clinical Trials as topic/ |  |
| 15. | Single Blind Method/ |  |
| 16. | or/1-15 |  |
| 17. | (clinical adj trial$).tw. |  |
| 18. | ((singl$ or doubl$ or treb$ or tripl$) adj (blind$3 or mask$3)).tw. |  |
| 19. | PLACEBOS/ |  |
| 20. | placebo$.tw. |  |
| 21. | randomly allocated.tw. |  |
| 22. | (allocated adj2 random$).tw. |  |
| 23. | or/17-22 |  |
| 24. | 16 or 23 |  |
| 25. | case report.tw. |  |
| 26. | letter/ |  |
| 27. | historical article/ |  |
| 28. | or/25-27 |  |
| 29. | 24 not 28 |  |
| 30. | traumatic brain injur*.mp. [mp=title, abstract, original title, name of substance word, subject heading word, keyword heading word, protocol supplementary concept word, rare disease supplementary concept word, unique identifier] |  |
| 31. | acute brain injur*.mp. [mp=title, abstract, original title, name of substance word, subject heading word, keyword heading word, protocol supplementary concept word, rare disease supplementary concept word, unique identifier] |  |
| 32. | brain injur*.mp. [mp=title, abstract, original title, name of substance word, subject heading word, keyword heading word, protocol supplementary concept word, rare disease supplementary concept word, unique identifier] |  |
| 33. | TBI.mp. [mp=title, abstract, original title, name of substance word, subject heading word, keyword heading word, protocol supplementary concept word, rare disease supplementary concept word, unique identifier] |  |
| 34. | neurological injur*.mp. [mp=title, abstract, original title, name of substance word, subject heading word, keyword heading word, protocol supplementary concept word, rare disease supplementary concept word, unique identifier] |  |
| 35. | Brain Injuries/ |  |
| 36. | or/30-35 |  |
| 37. | intensive insulin therapy.mp. [mp=title, abstract, original title, name of substance word, subject heading word, keyword heading word, protocol supplementary concept word, rare disease supplementary concept word, unique identifier] |  |
| 38. | Insulin/ |  |
| 39. | Glucose/ |  |
| 40. | Hypoglycemic Agents/ |  |
| 41. | intensive insulin.mp. [mp=title, abstract, original title, name of substance word, subject heading word, keyword heading word, protocol supplementary concept word, rare disease supplementary concept word, unique identifier] |  |
| 42. | or/37-41 |  |
| 43. | 29 and 36 and 42 |  |
| 44. | ICU.mp. [mp=title, abstract, original title, name of substance word, subject heading word, keyword heading word, protocol supplementary concept word, rare disease supplementary concept word, unique identifier] |  |
| 45. | intensive care.mp. [mp=title, abstract, original title, name of substance word, subject heading word, keyword heading word, protocol supplementary concept word, rare disease supplementary concept word, unique identifier] |  |
| 46. | critical care.mp. [mp=title, abstract, original title, name of substance word, subject heading word, keyword heading word, protocol supplementary concept word, rare disease supplementary concept word, unique identifier] |  |
| 47. | Critical Care/ or Intensive Care Units/ |  |
| 48. | 36 or 44 or 45 or 46 or 47 |  |
| 49. | 29 and 42 and 48 |  |
| 50. | (Intensive insulin therapy and pentastarch resuscitation in severe sepsis).m_titl. |  |
| 51. | 49 or 50 |  |
| 52. | critically ill.mp. [mp=title, abstract, original title, name of substance word, subject heading word, keyword heading word, protocol supplementary concept word, rare disease supplementary concept word, unique identifier] |  |
| 53. | 48 or 52 |  |
| 54. | 29 and 42 and 53 |  |
| 55. | 50 or 54 |  |
| 56. | limit 55 to (humans and "all adult (19 plus years)") |  |

**Cochrane**

#1 traumatic brain injur*:ti,ab,kw (Word variations have been searched)

#2 acute brain injur*:ti,ab,kw (Word variations have been searched)

#3 brain injur*:ti,ab,kw (Word variations have been searched)

#4 TBI:ti,ab,kw (Word variations have been searched)

#5 neurological injur*:ti,ab,kw (Word variations have been searched)

#6 MeSH descriptor: [Brain Injuries] explode all trees

#7 MeSH descriptor: [Brain Injuries] explode all trees

#8 "ICU":ti,ab,kw (Word variations have been searched)

#9 "critical care":ti,ab,kw (Word variations have been searched)

#10 intensive care:ti,ab,kw (Word variations have been searched)

#11 critically ill:ti,ab,kw (Word variations have been searched)

#12 MeSH descriptor: [Critical Care] explode all trees

#13 #1 or #2 or #3 or #4 or #5 or #6 or #7 or #8 or #9 or #10 or #11 or #12

#14 intensive insulin therapy:ti,ab,kw (Word variations have been searched)

#15 intensive insulin:ti,ab,kw (Word variations have been searched)

#16 MeSH descriptor: [Glucose] explode all trees

#17 MeSH descriptor: [Insulin] explode all trees

#18 MeSH descriptor: [Hypoglycemic Agents] explode all trees

#19 #14 or #15 or #16 or #17 or #18

#20 #13 and #19 in Trials

**Figure S1.** Network Meta-analysis map, with ‘tight’ control as the common reference group.

**Figure S2.** Forest plot for severe hypoglycaemia, summarised as risk difference, continuity correction zero.
